# A prospective observational study examining weight and psychosocial change in adolescent and adult eating disorder inpatients admitted for nutritional rehabilitation using a high-energy re-feeding protocol

**DOI:** 10.1186/s40337-024-01015-x

**Published:** 2024-05-14

**Authors:** Fiona Salter, Urvashnee Singh, Deborah Kerr, Yun Zhao, Emily Jeffery

**Affiliations:** 1 School of Population Health, Curtin University, Kent Street, GPO Box U1987, Perth, WA 6845 Australia; 2 Ramsay Clinic Hollywood, Hollywood Private Hospital, 95 Monash Avenue, Nedlands, WA 6009 Australia; 3 Curtin Health Innovation Research Institute, Curtin University, Kent Street, GPO Box U1987, Perth, WA 6845 Australia; 4 Present Address: Esus Centre, Centre of Excellence in the Treatment of Eating Disorders, 588, Hay Street, Subiaco, WA 6008 Australia

**Keywords:** Nutritional restoration, High-calorie, Anorexia nervosa, Bulimia nervosa, ARFID, Psychology

## Abstract

**Background:**

High-energy re-feeding protocols are increasingly utilised for nutritional rehabilitation in adolescents with anorexia nervosa (AN), however, concern persists that adults with AN may be at greater risk of developing complications. In addition, research on psychological outcomes of eating disorder (ED) inpatient treatment programs, and outcomes of high-energy protocols in avoidant restrictive food intake disorder (ARFID) and bulimia nervosa (BN), is limited. This study of an ED inpatient program using a high-energy protocol, compared changes in weight and psychosocial outcomes between adolescents and adults, and identified medical risk factors associated with deviation from the protocol.

**Method:**

This prospective observational study took place in a voluntary ED treatment program in a private hospital. Weight, height, and psychosocial questionnaires (ED Examination-Questionnaire, Depression Anxiety Stress Score, Clinical Impairment Assessment and AN/BN Stage of Change) were collected from consenting adolescents (16–20 years) and adults (> 20 years) on admission and discharge. Medical tolerance to the high-energy protocol was assessed daily. Independent samples t-tests and paired samples t-tests were applied to normally distributed data, and Mann–Whitney U tests and Wilcoxon signed-rank tests to skewed data. P-values < 0.05 were considered significant statistically.

**Results:**

Ninety-seven participants were recruited. The majority (n = 91, 94%) were female and most (n = 80, 83%) had AN. Forty-two (43%) were adolescents and 55 (57%) were adults. In participants with AN, weight change (Δ) was significant [median Δ 8.0 (interquartile range (IQR) 4.3) kg]. There was no difference in rate of weight change between adolescents and adults with AN [mean Δ 1.8 (standard deviation (SD) 0.5) kg/week vs. Δ 1.8 (SD 0.6) kg/week; p = 0.841, respectively]. One (1%) participant with AN did not tolerate the high-energy protocol due to oedema. Participants achieved positive change in psychosocial questionnaire scores (p < 0.001) after the the specialist ED program, with no difference between adolescents and adults (p > 0.05).

**Conclusions:**

This voluntary ED treatment program using a high energy re-feeding protocol was effective in achieving positive weight and psychological change for adolescents and adults with minimal adverse events. This indicates that the specialist ED program has both nutritional and psychological benefits.

**Supplementary Information:**

The online version contains supplementary material available at 10.1186/s40337-024-01015-x.

## Abstract (plain language summary)

Nutritional recovery of adolescent inpatients with anorexia nervosa (AN) can be safely and effectively carried out using high-energy feeding protocols. However, not enough research has been done to support the use of these protocols in adults with AN, or people with avoidant restrictive food intake disorder (ARFID) and bulimia nervosa (BN). The psychological effects of using high-energy protocols for people with eating disorders (EDs) are also rarely reported. We aimed to find out a) if our high-energy re-feeding protocol is effective for adolescents and adults with AN, and for people with other EDs; b) are there any medical side-effects of re-feeding that require the feeding protocol to change; c) do psychosocial measures of health change from admission to discharge; and d) are there differences in weight or psychosocial change between adolescents and adults? We found that for adolescents and adults with AN, weight increases were the same when using the high-energy protocol. The protocol was changed for only one participant with AN, who experienced oedema. Both adolescents and adults had positive improvements across all psychosocial questionnaires. The results of this research will help to guide clinicians and researchers on safe and effective care for people with EDs in voluntary treatment settings.

**Supplementary Information:**

The online version contains supplementary material available at 10.1186/s40337-024-01015-x.

## Background

People with eating disorders (EDs) have one of the highest mortality rates of all those living with mental illness, with anorexia nervosa (AN) carrying the highest risk of all EDs [1–3]. Prolonged periods of inadequate or imbalanced nutritional intake in people with EDs result in the development of starvation syndrome and its inherent consequences including increased disability, lost productivity, and social isolation [2]. For those with the highest medical and psychiatric risk, hospital admission to a specialist ED treatment centre is recommended to achieve medical stabilisation and nutritional restoration [4]. Multidisciplinary inpatient treatment, provided within a specialist ED program, is also an opportunity to effect psychological change [5].

Provision of regular and adequate nutrition to support positive weight change and limit the effects of starvation on the brain is a foundational step in treatment of and recovery from EDs [6, 7]. The current guidelines for re-feeding malnourished people with AN recommend starting with low energy intakes and increasing cautiously. However, these guidelines contribute to prolonged starvation and long hospital admissions [8, 9]. Recently, more rapid re-feeding, using higher energy re-feeding protocols, has been shown to be safe and effective for adolescents and young adults with severe malnutrition, and the use of high energy re-feeding protocols for nutritional restoration is becoming standard practice in these age groups [10–12].

The data available to inform best practice in adult ED populations and in relatively new ED diagnostic subtypes, such as avoidant restrictive food intake disorder (ARFID) and atypical AN, are lacking [13–15]. A persisting concern has been that adults with AN presenting with moderate to severe malnutrition may be more predisposed to developing potentially life-threatening electrolyte derangements [16]. In addition, frequent psychiatric comorbidities found in adult patients may complicate their medical care [17]. A review of studies investigating re-feeding syndrome outcomes in adults with EDs re-fed using higher energy intakes, found limited evidence of detrimental effects [18]. Emerging research also indicates that with close medical monitoring and with electrolyte supplementation, higher rates of re-feeding may be applied to extremely malnourished adults with AN [18]. Further research is recommended to identify risk factors for developing re-feeding syndrome in malnourished adults who are re-fed using high-energy re-feeding protocols, in order to implement timely and safe treatment [19].

While nutritional and medical outcomes of inpatient treatment for EDs are commonly reported, there is a lack of research available on the psychological outcomes [11, 12, 20]. Positive psychological change during ED inpatient treatment, by means of reversal of starvation syndrome through nutritional rehabilitation and psychological group therapy, could be advantageous in longer term recovery. A recent systematic review of outcomes of inpatient psychological treatments for adolescents with EDs found a paucity of high-quality studies, with conflicting results in relation to symptom and motivational change [20]. Further research is required to determine specific factors contributing to positive inpatient psychological change, and to identify whether ED inpatient programs developed for adults are as effective for adolescents who have different psychosocial needs [21].

In this study of patients admitted to a specialist ED program using a high-energy re-feeding protocol, the aim was to investigate weight, safety, and psychological outcomes. Objectives were to measure weight change and rate of weight change from admission to discharge and compare differences in weight change between adolescents and adults with AN and between the different ED diagnoses; to identify medical risk factors that require a deviation from the re-feeding protocol; and to describe changes in psychosocial measures of health and compare the differences in psychosocial change between adolescents and adults.

## Methods

### Study setting

The study took place in a 10-bed inpatient specialist ED Program within a 101-bed inpatient mental health unit in a large private hospital in Western Australia. The primary aim of the program is to provide nutritional rehabilitation, including positive weight change, for patients who are at medical risk or who have been unable to make progress with their nutritional rehabilitation in the community. Individuals with bulimia nervosa (BN) are also admitted for support towards regular eating and management of compensatory behaviours. The multidisciplinary ED program team consisted of a single psychiatrist clinical lead, psychiatry registrar, junior medical officer, dietitian, clinical nurse specialist, mental health nurses, clinical psychologists, an occupational therapist, and peer support worker. Admission criteria for the ED program (Supplementary Fig. 2) were: primary diagnosis of either AN, ARFID or BN, aged ≥ 16 years, body mass index (BMI) > 13 kg/m^2^, meeting criteria for an inpatient admission as a result of medical and/or psychiatric risk according to the Western Australian Eating Disorders Outreach and Consultation Service criteria [22], and the capacity to engage in voluntary ED treatment as assessed pre-admission by the nurse coordinator and/or admitting psychiatrist.

### Study sample

All patients who met the ED program admission criteria and who were admitted to the inpatient ED program between 1 April 2021 and 7 June 2022 were eligible for inclusion in the study. Exclusion criteria were (a) re-admission to the ED program during the study period, (b) unable to give informed consent, (c) inability to read or understand English or (d) discharge prior to completing the first twelve days of the re-feeding protocol. Written informed consent was required for this study. This study was approved by the Ramsay Health Care WA/SA Human Research Ethics Committee (HREC 2056) and Curtin University Ethics Committee (Reciprocal ethics approval number HRE2022-0033).

### Study design

**Fig. 1:**
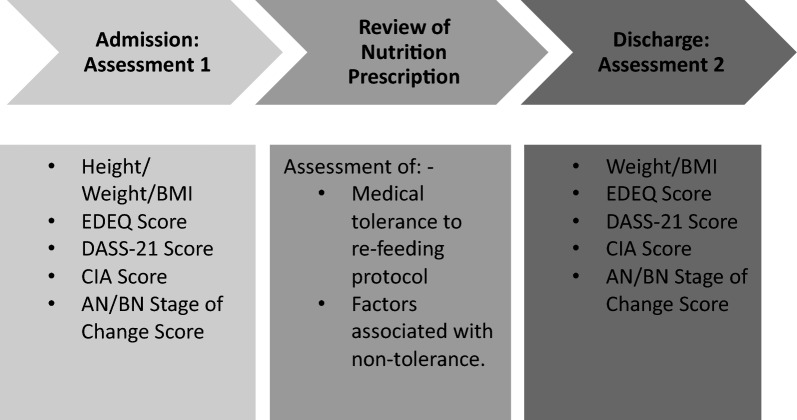
Study design. EDEQ Eating Disorders Examination Questionnaire, DASS-21 Depression Anxiety Stress Scale, CIA Clinical Impairment Assessment, AN/BN Stage of Change Scores

This was a prospective observational cohort study. Participants underwent anthropometric and psychosocial assessment within two days of admission and discharge (Fig. 1). Participant medical tolerance to the re-feeding protocol was assessed by the multidisciplinary team during daily medical and psychological reviews and clinical meetings. Briefly, blood biochemistry was measured on admission and then three times weekly for 14 days. Further details on the type and frequency of medical monitoring during the admission is provided in Supplementary Table 4.

### Demographic and medical characteristics

Participant demographic and medical characteristics were collected by the primary researcher. The data collected were primary ED diagnosis, duration of illness, co-morbidities, age, gender, length of hospital stay, and the presence of medical risk factors (hypophosphatemia, hypomagnesaemia, hypokalaemia, hyponatraemia, reduced thiamine levels and oedema) determined to require modification of the re-feeding prescription [23]. The treating psychiatrist determined co-morbidity by serial clinical interviews and mental state examinations during a patient's admission, integrated with psychiatric history and collateral history. Diagnosis of both primary and comorbid conditions were based on DSM-5 criteria [24]. Atypical AN was not differentiated from AN due to similar psychiatric and medical risk [15]. Length of stay was recorded as number of days from admission to discharge, including the day of discharge.

### Anthropometric measures

Participant height and weight were measured by trained nursing staff and BMI calculated by the dietitian. Height was measured to 0.1 cm on admission using a stadiometer. Weight was measured with participants wearing only a hospital gown to 0.1 kg on admission, then three times weekly until discharge, using calibrated weighing scales.

### Measures of eating disorder symptomology, mental health, quality of life and stage of change

Participants were given questionnaires by their nurse for self-completion on admission and within 24 h of discharge from hospital. The Eating Disorders Examination Questionnaire (EDE-Q) measures ED symptomology through 28 items using a 7-point likert scale. The EDE-Q is validated in older adolescents and adults with AN and BN and is widely used in clinical practice and research [25–27]. Questionnaires to measure disease severity in ARFID were not validated for use in research at the start of this study. The EDE-Q was deemed unsuitable and therefore not administered for patients with ARFID [28]. The Depression Anxiety Stress Scale (DASS-21) measures the emotional states of depression, anxiety and stress through 3 subscales within 21 items using a 4-point likert scale [29–31]. The DASS-21 was determined to be appropriate for use with AN, BN and ARFID patients due to its extensive use and evaluation in the ED and broader mental health field. The Clinical Impairment Assessment (CIA) questionnaire measures the severity of psychosocial impairment due to ED features through 16 items using a 4-point likert scale [32, 33]. The CIA was determined to be appropriate for use with all patients, including those with ARFID, as psychosocial impairment from ARFID has the potential to be similar to other EDs [24]. The AN and BN Stage of Change questionnaires (ANSOC and BNSOC) measure stage of change through 20 items using 5 statements relating to progressively increased readiness to change. The Stages of Change, or Transtheoretical Model, assesses an individual’s readiness to act on a new healthier behaviour [34]. The ANSOC and BNSOC questionnaires were considered unsuitable for use in patients with ARFID due to markedly different cognitive distortions in ARFID compared with AN and BN [24].

### Re-feeding protocol

A detailed description of the re-feeding protocol and the ED program supporting re-feeding is provided in the supporting information for this manuscript (Supplementary Table 1). Briefly, the dietitian assesses the patient’s current nutritional intake on admission (Supplementary Fig. 1), and if intake is assessed to be < 1700–1800 kcal/day (< 7100–7500 kJ/d), the nutrition prescription is either 1) 50% of each meal served and three 200 ml, 1.5 kcal/ml (6.3 kJ/ml) oral nutrition supplements, or 2) 50% of each meal served, 50% of each snack served and 600 ml of an overnight nasogastric feed. Protocol increments to 75% and 100% meals and snacks occur every 2 days. Regarding the 50–75% meal portions, a complete meal is plated and served from admission to visually reinforce patients’ understanding of a normal meal size. Patients are required to eat, at a minimum, the amount that corresponds with the protocol day. The amount of food left on the plate after the mealtime is recorded to assess whether patients have met their required target intake, and if not, to enable calculation of the bolus amount of enteral nutrition required (Supplementary Table 2). If current intake is assessed to be > 1700–1800 kcal/day (> 7100–7500 kJ/d), the nutritional prescription is for an additional 400–600 kcal (1700–2500 kJ), to prevent weight declining. For patients who experience ≥ 2 episodes of hypoglycaemia during the admission, a continuous nasogastric feed (NG) is prescribed. The initial phase of the protocol is the first 12 days, with standardised increments to nutritional intake being implemented every second day for twelve days. From day 13 onward, increments are based on the rate of positive weight change. For patients admitted primarily for support to manage ED compensatory behaviours such as those with BN, the goal intake is based on preadmission intake, taking into account binge and purge behaviours, with an initial goal intake of up to three meals and three snacks (Supplementary Table 3). A minimum target weight set at BMI > 20 kg/m^2^, or above and individualised for each patient, based on age, weight trajectory based on centile charts for adolescents, premorbid weight, weight history, medical stability and genetic factors is determined. For most patients the minimum target weight for hospital admission is set at BMI > 20 kg/m^2^. For some patients, a higher minimum BMI target is set based on individual factors listed including a higher premorbid weight. For adolescents aged 16–17 years, BMI > 20 kg/m^2^ corresponds to a BMI for age of between the 25th and 50th percentile on Centres for Disease Control growth charts [35]. The patient’s target BMI and rationale are discussed with the patient at the outset of the admission, highlighting that their healthy weight may well be higher than their discharge weight, and that this will be explored further during outpatient treatment. In our study, discharge weight was not included as an outcome measure to avoid inaccurate interpretations of the efficacy of the re-feeding protocol. While this ED program aims to achieve weight increase to a minimum BMI of 20 kg/m^2^ or above, patients are sometimes discharged prior to reaching their individual BMI goal for reasons including inability to comply with the program, medical or psychiatric instability necessitating transfer to a more appropriate setting or discharge against medical advice. Rates of weight and BMI change were therefore identified as primary outcome measures as we believe they more accurately capture the efficacy of the re-feeding protocol during hospital admission.

### Statistical methods

Change in weight/BMI, and change in EDE-Q, DASS-21, CIA and ANSOC or BNSOC scores from admission to discharge (i.e., outcomes on discharge-outcomes on admission) were calculated for patients in each of the ED diagnostic categories of AN, ARFID and BN. Only participants who completed both full admission and discharge questionnaires were included in the analysis. The paired samples t-test reporting mean [SD] (for normally distributed data) and Wilcoxon Signed Ranks Test reporting median [IQR] (for skewed data) were used to assess the change from admission to discharge. Differences between adolescent and adult participants with AN were calculated for weight and BMI on admission, length of stay in days, and rate of weight change in kg per week. Differences between adolescent and adult participants were calculated for each psychosocial measure of change. The independent samples t-test reporting mean [SD] (for normally distributed data) and the Mann–Whitney U Test reporting median [IQR] (for skewed data) was used to assess the difference between the two participant groups. The IBM SPSS Statistics for Windows (version 27, IBM Corporation, Somers, NY, USA) was used for data analysis with p values < 0.05 considered significant statistically.

## Results

### Participant characteristics, admission BMI and length of stay data

**Fig. 2:**
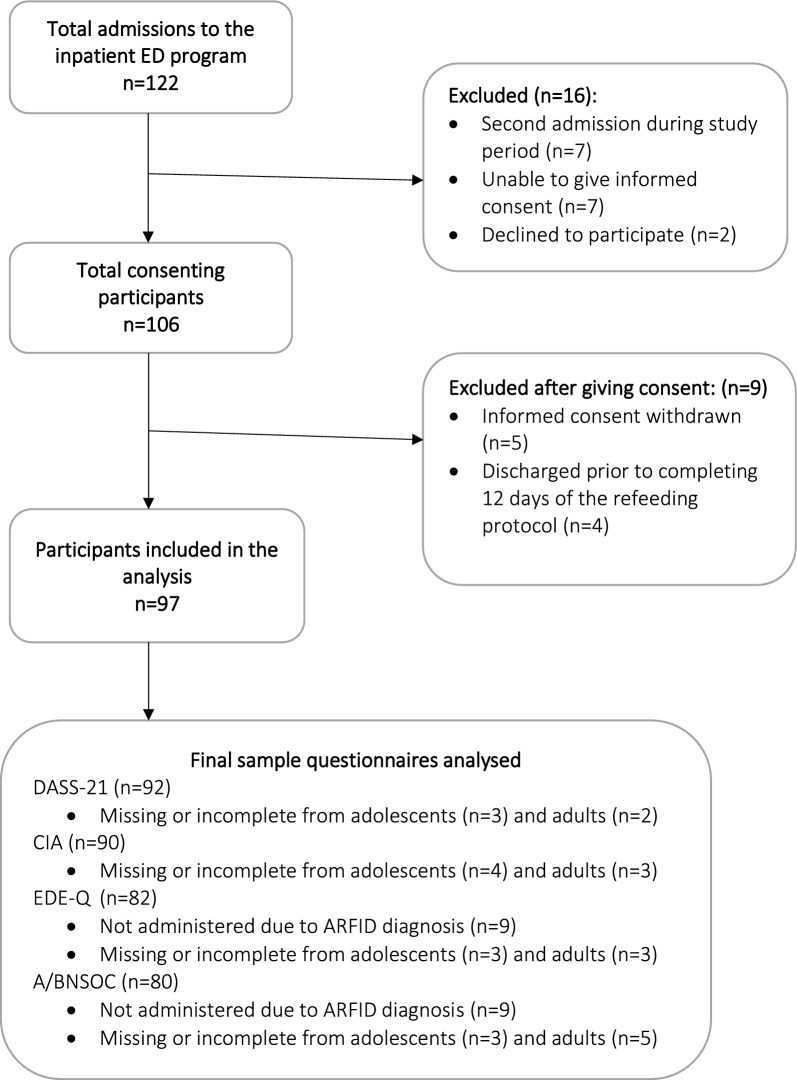
Study population—exclusions. EDEQ Eating Disorders Examination Questionnaire, DASS-21 Depression Anxiety Stress Scale, CIA Clinical Impairment Assessment, AN/BNSOC—AN/BN Stage of Change Scores

There were 122 patients admitted to the ED program during the study period, and of these patients, 97 were included in the current study (Fig. 2). Forty-two (43%) adolescents and 55 (57%) adults were included. Most participants were female (n = 91, 94%). Participant characteristics are summarised in Table 1. The majority of participants (n = 80, 83%) had a diagnosis of AN, primarily of the restrictive sub-type (n = 59, 60.8%) including four (4.1%) atypical AN, while nine participants (9.3%) had ARFID, and eight participants (8.2%) had BN. Generalised Anxiety Disorder was the most frequently diagnosed comorbidity in all age and ED diagnostic categories. There was a significant difference in duration of illness between adult and adolescent AN participants (p = 0.007) with adult AN patients having a longer illness duration. There were no significant differences in the mean admission BMI between adolescents and adults in the AN group (mean 18.6 [SD 2.1] and mean 18.1 [SD 3.2] kg/m^2^, respectively; p = 0.900). Two adults with AN and one adult with ARFID were classified as severely malnourished (BMI < 15 kg/m^2^). There was no significant difference in length of stay between adolescents and adults in the AN group (median 28 [IQR 13] days and median 31 [IQR 18] days, respectively; p = 0.730). Statistical tests were not performed to assess the differences in duration of illness, admission BMI or length of stay between adolescents and adults with BN or ARFID due to insufficient participant numbers.

**Table 1:** Description of study population

Description of study population (N = 97)							
Diagnosis	AN n = 80 (82.5%)		ARFID n = 9 (9.3%)			BN n = 8 (8.2%)	
Age category	Adolescents 16–20 years	Adults > 20 years	Adolescents 16–20 years		Adults > 20 years	Adolescents 16–20 years	Adults > 20 years
**n (%)**	36 (45)	44 (55)	4 (44)		5 (56)	2 (25)	6 (75)
**Age in years**							
Mean (SD)	17.7 (1.5)	28.1 (8)	18.8 (1.9)		32.6 (20.5)	19.0 (0)	35.7 (7.9)
**AN Subtype**							
Restrict	30 (83)	29 (66)	N/A		N/A	N/A	N/A
Binge-Purge	6 (17)	15 (34)	N/A		N/A	N/A	N/A
**Atypical AN**	1 (25)	3 (75)	N/A		N/A	N/A	N/A
**Gender**							
Female	34 (94)	43 (98)	2 (50)		4 (80)	2 (100)	6 (100)
Male	2 (6)	1 (2)	1 (25)		1 (20)	0 (0)	0 (0)
Non-Binary	0 (0)	0 (0)	1 (25)		0 (0)	0 (0)	0 (0)
**Psychiatric Comorbidity**							
Anxiety	23 (64)	21 (48)	4 (100)		4 (80)	2 (100)	3 (50)
OCD	1 (3)	5 (11)	0 (0)		1 (20)	0 (0)	0 (0)
Depression	6 (17)	7 (16)	1 (25)		0 (0)	0 (0)	1 (17)
BP	0 (0)	2 (5)	0 (0)		0 (0)	0 (0)	1 (17)
PD	7 (19)	14 (32)	0 (0)		0 (0)	0 (0)	1 (17)
Substance use disorders	2 (6)	4 (9)	1 (25)		0 (0)	0 (0)	1 (17)
Schizoaffective Disorder	0 (0)	1 (2)	0 (0)		0 (0)	0 (0)	0 (0)
ADHD	1 (3)	1 (2)	0 (0)		0 (0)	0 (0)	0 (0)
ASD	1 (3)	0 (0)	1 (25)		0 (0)	0 (0)	0 (0)
PTSD	6 (17)	6 (14)	0 (0)		0 (0)	0 (0)	0 (0)
SSD	1 (3)	2 (5)	0 (0)		1 (20)	0 (0)	0 (0)
PCGD	0 (0)	1 (2)	0 (0)		0 (0)	0 (0)	0 (0)
**Medical comorbidity**							
T1DM	2 (6)	0 (0)	0 (0)		0 (0)	0 (0)	1 (17)
Coeliac Disease	0 (0)	0 (0)	1 (25)		0 (0)	0 (0)	0 (0)
Bariatric surgery	0 (0)	2 (5)	0 (0)		0 (0)	0 (0)	1 (17)
Hypothyroid	0 (0)	0 (0)	1 (25)		0 (0)	0 (0)	0 (0)
Pregnancy	0 (0)	1 (2)	0 (0)		0 (0)	0 (0)	0 (0)
**Duration of illness in years**							
Median (IQR)	2.5 (1)	6(10)	11 (10)		3 (10.5)	5 (-)	19(27)
p value	.007**		^			^	
**Admission BMI** (**kg/m**^**2**^**) and difference between adolescents and adults**							
Mean	18.6	18.1	17.1		16.2	29.5	28.0
SD	2.1	3.2	2.6		1.2	6.1	3.0
Min–max	15.8–23.5	13.4–29.6	15.3–20.9		14.9–17.5	25.2–33.8	24.1–32.8
p value	.900		^			^	
**Degree of malnutrition n (%)**							
Mild/moderate	36 (100)	42 (95)	4 (100)	4 (80)		N/A	N/A
Severe	0 (0)	2 (5)	0 (0)	1 (20)		N/A	N/A
**Length of stay** (**days) and difference between adolescents and adults**							
Median	28	31	25		38	31	18
IQR	13	18	10		13	0	4
p value	.073		^			^	

AN—Anorexia Nervosa, ARFID—Avoidant Restrictive Food Intake Disorder, BN—Bulimia Nervosa, OCD—Obsessive Compulsive Disorder, BP—Bipolar Disorder, PDs—Personality Disorders, ADHD—Attention Deficit Hyperactivity Disorder, ASD—Autism Spectrum Disorder, PTSD—Post Traumatic Stress Disorder, SSD—Somatic Symptom Disorder, PCGD—persistent complex bereavement disorder, T1DM—Type 1 Diabetes Mellitus. P Values define differences in Duration of Illness, Admission BMI and Length of Stay between adolescents and adults *P value < 0.05 significant **P value < 0.01 extremely significant. ^ statistical tests not performed due to insufficient participant numbers

### Changes in body weight

Weight and BMI changes from admission to discharge for all patients and differences between adults and adolescents with AN are summarised in Table 2. In all participants with AN and ARFID, total weight increased significantly from admission to discharge (AN group: median Δ 8.0 [IQR 4.3]kg; p < 0.001 and ARFID group median Δ 8.5 [IQR 6.8] kg; p = 0.008). In both adolescents and adults with AN, weight increased significantly from admission to discharge (median Δ 6.9 [IQR 3.3] kg vs. median Δ 9.0 [IQR 4.1] kg respectively; p < 0.001) and there was no statistically significant difference between the two age categories (p = 0.052). Weight increases over the admission were significant in adults with ARFID (median Δ 8.5 [IQR 6.7] kg, p = 0.043) but not significant for adolescents (median Δ 7.5 [IQR 6.2] kg, p = 0.068). Weight change was not significant for participants with BN, nor for adolescent and adult participants with BN (median Δ 0.7 [IQR 0.9] kg vs. median Δ 0.4 [IQR 0.5] kg, p = 1.000, respectively). Statistical tests were not performed to assess differences in change in body weight between adolescents and adults with BN or ARFID due to insufficient participant numbers.

**Table 2:** Weight and BMI change from admission to discharge and differences between adults and adolescents

Diagnosis	Age category	Weight change (discharge weight—admission weight) kg			BMI change (discharge BMI—admission BMI) kg/m^2^			Difference between adults and adolescents			
								Total weight change	Rate of weight change kg/week		
		Median	IQR	P* value	Median	IQR	P* value	P^#^ value	Mean	SD	P^&^ value
AN	All	8.0	4.3	0.001	2.9	1.6	0.001				
	Adolescents	6.9	3.3	0.001	2.5	1.4	0.001	0.052	1.8	0.5	0.841
	Adults	9.0	4.1	0.001	3.2	1.5	0.001		1.8	0.6	
ARFID	All	8.5	6.8	0.008	2.9	2.5	0.008				
	Adolescents	7.5	6.2	0.068	2.4	2.6	0.068	^	1.9	0.7	^
	Adults	8.5	6.7	0.043	2.9	2.0	0.042		1.9	0.3	
BN	All	0.4	1.5	0.175	0.2	0.6	0.175				
	Adolescents	0.7	0.9	0.655	0.3	0.4	0.655	^	0.2	0.3	^
	Adults	0.4	0.5	0.249	0.2	0.3	0.279		0.2	0.5	

AN—Anorexia Nervosa, ARFID—Avoidant Restrictive Food Intake Disorder, BN Bulimia Nervosa

*Wilcoxon Signed Ranks Test

^#^Mann–Whitney U Test

^&^Independent samples t test; p value < 0.05 indicates statistically significant at 5% level

^Statistical tests not performed due to insufficient participant numbers

### Re-feeding methods and medical tolerance to the re-feeding protocol

**Fig. 3:**
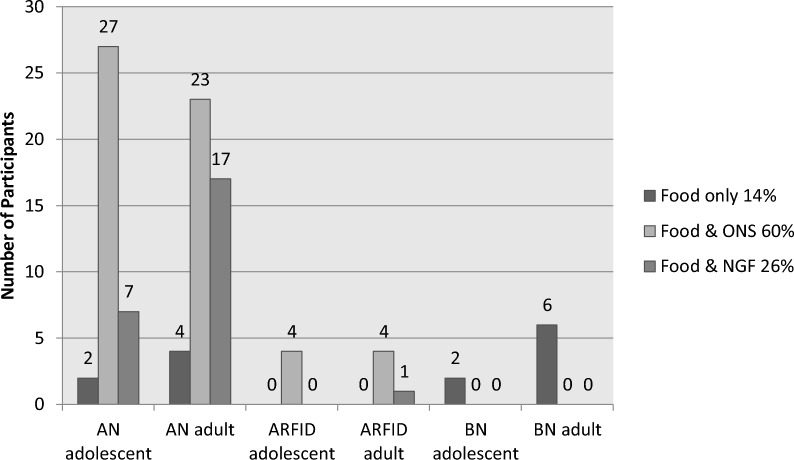
Re-feeding methods prescribed. AN—Anorexia Nervosa, ARFID—Avoidant Restrictive Food Intake Disorder, BN—Bulimia Nervosa, ONS—Oral Nutritional Support, NGF—Nasogastric Feed

The refeeding methods used are shown in Fig. 3. Oral re-feeding with food only, or with food and oral nutritional support was prescribed by the dietitian for 74% (n = 72) of all participants and a NG feed was prescribed for one quarter (n = 24) of participants. In AN, 19% (n = 7) adolescents and 31% (n = 17) of adult participants were prescribed a NG feed. One adult (11%) with ARFID was prescribed a NG feed. All (100%) participants with BN were re-fed using food alone.

Most (99%) participants did not develop medical complications and were re-fed according to the protocol. The re-feeding protocol was adjusted for one adult with AN (binge-purge sub-type) diagnosed with early features of refeeding syndrome, namely oedema associated with hypokalaemia and hyponatraemia. There were no other cases of re-feeding syndrome (hypophosphatemia, hypomagnesaemia, or reduced thiamine levels) identified in participants.

### Psychosocial questionnaire scores

Table 3 summarises changes in psychosocial questionnaire scores. The changes were calculated by subtracting the admission scores from the discharge scores. The EDE-Q, DASS-21 and CIA questionnaires define a lower score as a better health status, and therefore a negative change in the EDE-Q, DASS-21 and CIA scores indicates an improved health status. There was a significant negative change (p < 0.001) in the total score and subscale scores of the EDE-Q, DASS-21 and CIA for both adolescents and adults, indicating an improvement in the participants’ disease severity, states of depression, anxiety and stress, and severity of psychosocial impairment, from admission to discharge. There was no significant difference in the questionnaire scores change between adolescents and adults except the EDE-Q weight concern subscale for AN and BN participants. Adolescents with AN and BN showed a greater change towards improved health in their weight concern score than adults with AN and BN (mean Δ -1.9 [SD 1.6] vs. mean Δ -1.0 [SD 1.5], respectively; p = 0.012). A higher score of AN/BNSOC questionnaires indicates a better health status, hence a positive change in AN/BNSOC scores suggests an improvement in health accordingly. There was a significant positive change (p < 0.001) in the total score of AN/BNSOC for both adolescents and adults, indicating an improvement in the participants’ readiness to change from admission to discharge. There was no significant difference in the AN/BNSOC questionnaire scores change between adolescents and adults.

**Table 3:** Psychosocial questionnaire scores and differences between adolescents and adults

Scales	Adolescent scores							Adult scores							Difference in change between adolescent and adult
	Admit score		D/C score		Change (D/C—admit)			Admit score		D/C score		Score change (D/C—admit)			P^#^ value
	$\overline{x}$	σ	$\overline{x}$	σ	$\overline{x}$	σ	P* value	$\overline{x}$	σ	$\overline{x}$	σ	$\overline{x}$	σ	P* value	
**EDE-Q—AN & BN only**															
Restraint	4.2	1.5	0.8	0.9	− 3.4	1.4	< 0.001	4.5	1.5	1.1	1.2	− − 3.4	1.9	< 0.001	.957
Eating concern	4.2	2.3	1.4	1.1	− 2.8	2.3	< 0.001	5.2	2.7	1.8	1.2	− 3.4	2.7	< 0.001	.318
Shape concern	4.9	1.2	3.7	1.6	− 1.2	1.4	< 0.001	4.7	1.2	3.9	1.5	− 0.8	1.5	< 0.001	.162
Weight concern	4.6	1.3	2.7	1.7	− 1.9	1.6	< 0.001	4.4	1.3	3.4	1.6	− 1.0	1.5	< 0.001	.012
Global	4.5	1.3	2.1	1.1	− 2.4	1.3	< 0.001	4.7	1.3	2.5	1.1	− 2.1	1.4	< 0.001	.493
**DASS-21—AN, BN & ARFID**															
Depression	14.7	3.9	8.2	4.8	− 6.6	4.5	< 0.001	12.5	5.2	7.9	6.1	− 4.6	6.2	< 0.001	.095
Anxiety	11.9	5.1	7.3	4.3	− 4.6	4.9	< 0.001	9.8	4.6	7.0	5.1	− 2.8	5.0	< 0.001	.092
Stress	13.9	4.5	9.2	4.0	− 4.7	5.0	< 0.001	13.2	4.8	9.0	4.8	− 4.2	5.5	< 0.001	.652
**CIA—AN, BN & ARFID**															
Total score	36.7	7.5	23.2	11.2	− 13.5	11.5	< 0.001	36.8	7.2	22.0	11.5	− 14.9	11.2	< 0.001	.564
**ANSOC/BNSOC—AN & BN only**															
Total score	2.4	0.6	3.7	0.8	1.3	0.7	< 0.001	2.4	0.6	3.6	0.8	1.2	0.7	< 0.001	.628

Admit—Admission, D/C—Discharge EDEQ Eating Disorders Examination Questionnaire, DASS-21 Depression Anxiety Stress Scale, CIA Clinical Impairment Assessment, AN/BN Stage of Change Scores, $\overline{x}$—mean, σ—standard deviation

*Paired samples t test

^#^Independent samples t test; P value < 0.05 statistically significant, P value < 0.001 highly statistically significant

## Discussion

This prospective observational study of patients admitted to a voluntary ED treatment program aimed to compare changes in weight and psychosocial outcomes between adolescents and adults with AN, and between different ED diagnoses, and identify medical risk factors associated with a deviation from the high-energy re-feeding protocol. We found that the high-energy re-feeding protocol affected positive weight change for participants with AN and ARFID with a very low rate of adverse effects. Only one severely malnourished participant with AN (binge-purge subtype) required deviation from the protocol due to oedema. There was no difference in rate of weight change and length of hospital stay between adolescents and adults with AN. Furthermore, a significant positive psychological change was measured between admission and discharge for all participants, which was similar in adolescents and adults with EDs.

### Rate of weight change and energy prescription

The high-energy re-feeding protocol used in our study provided sufficient energy to optimise rates of positive weight change in adolescents and adults with EDs. Utilising our protocol, adolescents, and adults with AN and ARFID admitted at a similar BMI had a similar mean rates of weight change of 1.8 and 1.9 kg per week, respectively. This was likely due to most adolescents and adults in our study adhering to the re-feeding protocol and ongoing individualised measures implemented to support optimal weight change for the duration of their admission. These results are consistent with a study using a similar meal-based high-energy re-feeding protocol to re-feed adults with mixed EDs which found mean rates of weight change of 1.85 kg per week [36]. Previous studies report a range of “higher calorie” starting rates from 1500 kcal/day (6300 kJ/day), increasing by 500 kcal (2100 kJ) every 48 h [37], to 2000 kcal/day (8400 kJ/day) increasing by 200 kcal/day (800kJ/day)[12] Goal energy intakes also differ in previous studies and are commonly standardised, for example, to 3000 kcal/day (12,600 kJ/day) [38] or based on estimated energy requirements determined from predictive Eqs. (12). Energy prescriptions in our re-feeding protocol exceeded these amounts, with starting rates between 1700–2100 kcal/day (7100–8800 kJ/day), increasing by 400–600 kcal/day (1700–2500 kJ/day) every 48 h until goal energy intake 3700–3900 kcal/day (15,500–16300 kJ/day) were reached, after which time energy intake was individualised in order to achieve the goal rate of weight change of > 1.5 kg/week. Malnourished individuals, when re-fed, differ in their degree of hypermetabolism and activity despite support to manage compensatory activity during the admission. Therefore, individualised energy prescriptions, such as those used in our study, are required to support ongoing optimal rates of weight change beyond the initial re-feeding period.

### Safety of the re-feeding protocol

Ninety nine percent of participants in this study did not develop medical complications and tolerated the meal-based high-energy re-feeding protocol. There was one occurrence of oedema associated with electrolyte changes in a severely malnourished (BMI < 15 kg/m^2^) participant, however there were no episodes of hypophosphatemia, hypomagnesaemia, or low thiamine levels. This indicates that the protocol is feasible and safe for adults, when administered with concurrent prophylactic phosphate supplementation and close medical monitoring, to identify and treat any early decline in these markers of re-feeding syndrome. These results are consistent with other research in adults where high-energy re-feeding has been accomplished safely [19, 39]. Together with our research, these results challenge the appropriateness of the currently recommended low energy re-feeding guidelines for adults with AN. Notably, only three adult participants (3%) in our study were classified as severely malnourished (BMI < 15 kg/m^2^), due to admission criteria selecting people BMI > 13 kg/m^2^ who are ready to engage in treatment. This should be taken into consideration when interpreting the safety of the protocol, as a lower BMI on admission has been associated with a higher risk of re-feeding hypophosphataemia and oedema [40, 41].

### Length of stay and target weight

In our study, median length of stay for adolescents and adults with AN and ARFID was 28 and 31 days and 25 and 38 days, respectively. The median BMI increase during inpatient admission in all participants with AN and ARFID was 2.9 kg/m^2^. These results are consistent with a similar specialised mixed ED program using meal-based high-energy re-feeding, where mean length of stay was 39 days with a mean BMI increase of 3.0 kg/m^2^ [36]. Although average length of stay ranges from three to five weeks in these studies, weight change during hospitalisation, to > 85% expected body weight, is associated with a higher likelihood of maintained weight at one year [42] and decreases the probability of relapse and rehospitalisation [43]. In our study, malnourished participants minimum target weight was set at BMI > 20 kg/m^2^, or above and individualised based on age, weight trajectory based on centile charts for adolescents, premorbid weight, weight history, medical stability and genetic factors. Re-feeding protocols used to facilitate medical stabilisation without reaching an individual’s minimum target weight may contribute to prolonged illness through increased relapse and repeat admissions in some individuals, with associated higher costs [44, 45]. The use of high-energy re-feeding protocols, such as ours, demonstrate that optimal weight change can be achieved within an acceptable time frame in patients with AN and ARFID who meet medical risk indicators for admission or who are unable to progress with nutritional rehabilitation in the community. We also acknowledge the evidence for effectively reducing length of hospitalisation and costs of treatment with outpatient treatments, such as FBT and ED day program treatment, and recognise that the choice of treatment setting may be dependent upon the availability of different treatments within specific geographical areas [46, 47].

### Psychological change

In our study, adolescents, and adults with EDs showed a significant reduction in ED symptomology, depression, anxiety, stress and clinical impairment from their ED, and progression in stage of change over their admission. Most research on high-energy re-feeding of adult inpatients with EDs has focused on weight and treatment safety outcomes while psychological outcomes have been lacking [11, 12, 20], however in a small study of adolescent females who underwent high-energy re-feeding, significant reductions in depression and anxiety scores were found [48]. This study also reported significant positive change in ED symptomology, with the global EDE-Q score and eating concern sub-score (p < 0.05), and the restraint sub-scores (p < 0.01). Exploring the reasons for improvement in psychosocial outcomes was outside of the scope of the current study, however it is plausible that both the reversal of starvation syndrome and the psychological therapy provided by the specialist ED program (Supplementary Table 5), through repair of physiological, emotional, cognitive, and social functioning, could have contributed to these outcomes [23]. Further research investigating the factors contributing to improved psychosocial functioning is required to optimise ED inpatient treatment outcomes.

In our study, adolescents with AN and BN had a greater improvement in the EDE-Q weight concern sub-score compared with adults with AN and BN. To our knowledge, the difference between adolescent and adult psychological change during high-energy re-feeding has not been reported. Notably, the duration of illness was also significantly longer in adults than adolescents. Imaging data has shown greater brain structural differences in adult women with AN who were only partially weight restored, compared to those who had achieved sustained weight restoration when compared to healthy controls [7]. The study suggested that achieving and maintaining full weight restoration is important to diminish the possible neurobiological consequences of starvation associated with AN. It is possible that the longer duration of illness and consequent increased exposure to starvation in adults in our study may have had been a factor influencing clinical outcome, demonstrated by less improvement in the weight concern psychological test score at discharge. Further research is required to understand how neurophysiological changes affect nutritional and psychosocial outcomes.

## Limitations

The admission criteria to our ED program are intended to select patients with EDs who would benefit most from this treatment setting, therefore results may not be widely generalisable. Participants were mostly mild and moderately malnourished and therefore are not reflective of severely malnourished ED populations. The pre-requisite for patients admitted to our ED program, to be able to engage in treatment and consent to restore weight to a minimum target, would have likely selected participants with greater readiness to change. This may account for the associated increased treatment engagement and decreased ED symptomology. In this study, the reasons patients discharged early from the program was not collected but it is acknowledged that reasons for early discharge from the program, may be relevant to treatment tolerability. Pharmacological and behavioural management of anxiety, depression, and the side effects of re-feeding, as well as a positive therapeutic alliance, may also have contributed to improvement in psychosocial measures of health. Our study recruited sufficient adults and adolescents as well as participants with AN to draw meaningful conclusions, however, low numbers of participants with ARFID and BN limit conclusions that can be drawn from these groups. The numbers of people with ARFID and BN were, however, similar to those reported in other studies [14, 38, 49]. People with ARFID may be under-identified for hospital treatment due to limited awareness by health professionals and limited evidence-based assessment and treatment models [28]. People with BN are less likely to meet hospital admission criteria and therefore most are managed in the community [23]. In addition, new guidelines re-classify re-feeding syndrome as percentage decrease in electrolyte levels within the first 5 days of re-feeding, as opposed to the standard cut off values used in this study and may have diagnosed more participants with re-feeding syndrome [50]. It is unknown whether this would have resulted in a requirement for the re-feeding protocol to change, however, close medical monitoring and management in this ED program would likely have facilitated continued medical tolerance of the re-feeding protocol. In our study, Atypical AN was not differentiated from AN as the characteristics of patients with Atypical AN generally do not differ significantly from those with AN except for their current weight. In the authors view, making this distinction contributes to the weight bias that people with A-AN commonly experience in healthcare settings [51]. Since the primary physiological outcome measure in our study was rate of weight change and not final weight, and since patient weight targets were individualised, the results were not affected by the decision to combine AN with A-AN.

## Strengths

This study has several strengths. The prospective cohort study design contributed to a more complete data collection. For example, we had 100% completion of population descriptors and anthropometric data and 93% completion of psychosocial questionnaires. All admissions to the specialist ED program during the study period were eligible for inclusion in the study, with the exception of those that met exclusion criteria. This would contribute to a reduction in selection bias in this study. A limitation of previous studies is that they have often reported on AN alone or on outcomes of mixed EDs grouped together [11, 19, 36, 52]. Our study compared outcomes for adolescents and adults and different ED diagnoses, providing greater insight into the characteristics of each sub-group. This study also provided detailed methodology which includes a description of the high-energy re-feeding protocol and the ED treatment program, which enables replication of the study.

## Conclusion

The high-energy re-feeding protocol implemented in this prospective observational study affected significant positive weight change in participants with AN with low rates of adverse effects. There was no difference in the rate of weight change between adolescents and adults. Overall, participants experienced significant positive psychological change from admission to discharge. Similar improvements were demonstrated in both adolescents and adults. This indicates that the specialist ED program used in this voluntary treatment setting has both nutritional and psychological benefits. While reported adverse events were uncommon, clinicians should be aware that severely malnourished adults may require adjustments to high-energy re-feeding protocols to prevent re-feeding syndrome.

### Supplementary Information

Supplementary file 1 (DOCX 109 KB)

Supplementary file 2 (pdf 36 KB)

Supplementary file 3 (pdf 68 KB)

## Data Availability

The datasets used and/or analysed during the current study are available from the corresponding author on reasonable request.

## Abbreviations

AN: Anorexia Nervosa
ED: Eating Disorder
ARFID: Avoidant Restrictive Food Intake Disorder
BN: Bulimia Nervosa
BMI: Body Mass Index
EDE-Q: Eating Disorder Examination Questionnaire
DASS-21: Depression Anxiety Stress Scale – 21 questions
CIA: Clinical Impairment Assessment questionnaire
ANSOC: Anorexia Stage Of Change questionnaire
BNSOC: Bulimia Nervosa Stage Of Change questionnaire
