# Correction: A prospective observational study examining weight and psychosocial change in adolescent and adult eating disorder inpatients admitted for nutritional rehabilitation using a high-energy re-feeding protocol

**DOI:** 10.1186/s40337-024-01085-x

**Published:** 2024-08-21

**Authors:** Fiona Salter, Urvashnee Singh, Deborah Kerr, Yun Zhao, Emily Jeffery

**Affiliations:** 1 School of Population Health, Curtin University, Kent Street, GPO Box U1987, Perth, WA 6845 Australia; 2 Ramsay Clinic Hollywood, Hollywood Private Hospital, 95 Monash Avenue, Nedlands, WA 6009 Australia; 3 Curtin Health Innovation Research Institute, Curtin University, Kent Street, GPO Box U1987, Perth, WA 6845 Australia; 4 Esus Centre, Centre of Excellence in the Treatment of Eating Disorders, 588, Hay Street, Subiaco, WA 6008 Australia

**Correction to: Journal of Eating Disorders (2024) 12:58**

10.1186/s40337-024-01015-x

Following the publication of the Original Article, the author reported errors in the 2nd, 3rd, 4th, and 5th authors’ affiliations.

Incorrect:

Urvashnee Singh^1,^^4^, Deborah Kerr^2,^^3^, Yun Zhao^2^ and Emily Jefery^2^^*^.

Correct:

Urvashnee Singh^2,^^4^, Deborah Kerr^1,^^3^, Yun Zhao^1^ and Emily Jeffery^1^^*^.

The affiliations have been updated above.

The Original Article has been corrected.

