# Supplementary material for: A prospective observational study examining weight and psychosocial change in adolescent and adult eating disorder inpatients admitted for nutritional rehabilitation using a high-energy re-feeding protocol
Source: J Eat Disord. 2024 May 14;12:58. doi: 10.1186/s40337-024-01015-x (PMC11094855; doi:10.1186/s40337-024-01015-x)
Supplement: Supplementary file 1 — (DOCX 109 KB) [file 40337_2024_1015_MOESM1_ESM.docx]

**Supporting information for: A Prospective Observational Study Examining Weight and Psychosocial Change in Adolescent and Adult Eating Disorder Inpatients admitted for Nutritional Rehabilitation using a High-Energy Re-feeding Protocol**

**Supplementary Methods**

**Supplementary References**

**Supplementary Table S1 Refeeding Protocol**

**Supplementary Table S2 Bolus 1.5 kcal/ml (6.3 kJ) ONS volumes for missed meals and snacks**

**Supplementary Table S3 Baseline Daily Meal Plans for Females and Males**

**Supplementary Table S4. Summary of** **Inpatient ED Program Stages and Management**

**Supplementary Table S5. Summary of ED Inpatient groups**

**Supplementary Methods**

The methods described here are suggested for re-feeding malnourished older adolescents (>16 years) and adults (>20 years) with mixed EDs (AN, ARFID and BN) in a voluntary psychiatric treatment setting that provides medical monitoring and treatment. This 10-bed in-patient ED group program was developed at a private hospital in Western Australia.

This high energy re-feeding protocol provides individualised nutrition care for patients throughout their admission. The dietitian completes the nutrition assessment and formulates the nutrition care plan. The re-feeding protocol is implemented until initial goal energy intake is reached. After this the nutrition prescription is individualised to achieve weekly weight goals. Re-feeding is supported through multidisciplinary team interventions and the staged structure of the ED program.

**Nutrition Assessment**

On admission, the dietitian completes a detailed nutrition assessment of the patient including onset and severity of ED behaviors and their effects on food and fluid intake and weight; pre-admission energy intake; ED related compensatory behaviors such as bingeing, purging, laxative abuse, and over-exercising; and level of risk for re-feeding syndrome (1–3). Information pertaining to the assessment is sought from relevant healthcare professionals including primary healthcare physician, psychologist, outpatient or previous hospital dietitian and the patient’s family and relevant carers. A blind weight, unseen by the patient, is taken on the day of admission and the following morning to assess baseline weight as compensatory water loading or food and fluid intake immediately prior to admission may artificially increase weight. Height is also measured on the day of admission, and body mass index (BMI) for age is calculated. An example nutrition assessment form is shown in Supplementary ***Figure S1***.

**Re-feeding Protocol**

*Commencement of the Re-feeding Protocol*

Details of the re-feeding protocol are described in Supplementary ***Table S1***. In summary, patients admitted following a period of restricted energy intake of <1700 kcal/day (<7100 kJ/day), commence on Day 1 of the protocol, which provides 1700-1800 kcal/day (7100-7500 kJ/day). Patients with pre-admission energy intake >1700-1800 kcal/day are commenced one increment above the assessed intake on the protocol, to avoid weight loss resulting from a reduction of intake compared to pre-admission. For example, if a patient was consuming 1700-1800 kcal/day prior to admission, they would commence re-feeding at Day 3 of the protocol, which provides 2100-2250 kcal/day (8800-9400 kJ/day).

**Supplementary Table S1. Re-feeding Protocol**

|  | **Re-feeding with ONS**  **1.5 kcal/ml (6.3 kJ/ml)** | | | | **Re-feeding with NGF**  **1.5 kcal/ml (6.3 kJ/ml)** | | | |
| --- | --- | --- | --- | --- | --- | --- | --- | --- |
| **Day** | **Meals**  **%** | **Snacks**  **%** | **ONS**  **100%** | **Estimated kcals (kJ)** | **Meals**  **%** | **Snack**  **%** | **NGF**  **ml** | **Estimated kcals (kJ)** |
| **1 & 2** | 50 | 0 | 3 x 200ml | 1700-1800  (7100-7500) | 50 | 50 | 600 | 2000-2100  (8400-8800) |
| **3 & 4** | 75 | 0 | 3 x 200ml | 2100-2250  (8800-9400) | 75 | 75 | 600 | 2550-2700  (10700-11300) |
| **5 & 6** | 100 | 0 | 3 x 200ml | 2500-2700  (10500-11300) | 100 | 100 | 600 | 3100-3300  (13000-13800) |
| **7 & 8** | 100 | 3 | 3 x 200ml | 3100-3300  (13000-13800) | 100 | 100 | 600 | 3100-3300  (13000-13800) |
| **9 & 10** | 100 | 3 | 4 x 200ml | 3400-3600  (14200-15100) | 100 | 100 | 800 | 3400-3600  (14200-15100) |
| **11 & 12** | 100 | 3 | 5 x 200ml | 3700-3900  (15500-16300) | 100 | 100 | 1000 | 3700-3900  (15500-16300) |
| **Day 13 onwards individualise increments in meal plan based on weight restoration**  **Consider increments in meals, snacks, ONS, NGF**  **Consider reducing volume using 2 kcal/ml** | | | | | | | | |

Re-feeding protocol used for malnourished adults and older adolescents with EDs to support weight restoration of 1.5 – 2 Kg/week. NGF = nasogastric feed, ONS = oral nutrition support.

*Days 1-12 of Admission*

For the first twelve days of the inpatient stay, the proportion of meals and number of oral nutrition supplements (ONS) increase every second day ***(Supplementary Table S1)***. Incremental increases in energy intake are consistent with re-feeding guidelines for adolescents (4,5). Regarding the 50-75% meal portions, a complete meal is plated and served from admission to visually reinforce patients’ understanding of a normal meal size. Patients are required to eat, at a minimum, the amount that corresponds with the protocol day. The amount of food left on the plate after the mealtime is recorded to assess whether patients have met their required target intake, and if not, to enable calculation of the bolus amount of enteral nutrition required.

*Beyond Day 13 of Admission*

The re-feeding plan from Day 13 onward is individualised dependent on the patient’s rate of weight change. If weight increase is < 1.5-2.0 kg/week, energy intake is increased, in collaboration with the patient, by adding one >200 kcal (840 kJ) snack and one grain serve to the daily meal plan. If target rate of weight change is still not achieved, energy intake is increased by 200ml of a 1.5 kcal/ml (6.3 kJ) ONS or by the energy equivalent as food. Patients requiring larger volumes of ONS or who are unable to manage the volume of ONS required are offered 2.0 kcal/ml (8.4 kJ) ONS to maintain energy intake whilst reducing ONS volume. Once patients reach their minimum weight target set for the admission, the ONS drinks are gradually reduced from the meal plan by 200 ml on alternate days, with the aim of maintaining weight above the minimum weight target prior to discharge from hospital and transfer to the patients’ community team. The meal plan for home reflects the elevated metabolic rate that persists following re-feeding and patient’s meal plans are individualised to accommodate this. Meal plans at discharge are typically three main meals and four to five snacks.

*Weight Monitoring During Admission*

Blind weight measurements are recorded three times per week to identify early plateau or decline in weight and facilitate appropriate and timely intervention. Weight measurements are taken in the morning after voiding and prior to consuming food or fluid, and with the patient wearing only a hospital gown. If sub-optimal weight change is identified, possible causes are investigated, and an intervention is commenced. For example, hyper-metabolism leading to increased total energy expenditure is addressed by an increase in energy intake, while compensatory behaviors such as exercise or purging are discussed with patients to identify triggers for the behavior and measures to support patients to cease these behaviors. Oedema or constipation may require medical management and a review of increments to the individual re-feeding protocol whilst these issues are resolved. There may be no change in treatment if weight change over the week is adequate and no other cause for a weight plateau or small decline has been identified. The minimum target weight determined for the admission is based on several factors including age, weight trajectory based on centile charts for adolescents, premorbid weight, weight history, medical stability, and genetic factors. For most patients the minimum target weight for hospital admission is set at BMI > 20 kg/m^2^. For adolescents aged 16-17 years BMI > 20 kg/m^2^ corresponds to a BMI for age of between the 25^th^ and 50^th^ percentile on Centres for Disease Control growth charts (6). The patient’s target BMI and rationale are discussed with the patient at the outset of the admission, highlighting that their healthy weight may well be higher than their discharge weight, and that this will be explored further during outpatient treatment.

*Management of Missed Meals and Snacks*

Patients are given a bolus of an ONS immediately following a meal or snack if the patient has not achieved 100% of their goal intake. The bolus is offered orally but may be given via the nasogastric (NG) tube if this is already being used for overnight NG feeds. Compliance with eating meals and snacks is incentivised over ONS boluses by providing a bolus requirement being of a greater energy value than the meal and snack. For example, 400ml (or proportion) of a 1.5-kcal/ml ONS is given for missed main meals, and 200ml (or proportion) of a 1.5-kcal/ml ONS is given for missed snacks. The bolus chart used in practice and outlined in Supplementary ***Table S2*** shows the proportion of supplement required in relation to the proportion of meal or snack missed.

**Supplementary Table S2.** **Bolus 1.5 kcal/ml (6.3 kJ) ONS volumes for missed meals and snacks**

|  | **% of required food left uneaten** | **100** | **75** | **50** | **25** | **0** |
| --- | --- | --- | --- | --- | --- | --- |
| **Bolus ml for Required Intake 100%** | **Main Meal** | 400 | 300 | 200 | 100 | 0 |
|  | **Snack** | 200 | 150 | 100 | 50 | 0 |
| **Bolus ml for Required**  **Intake 75%** | **Main Meal** | 300 | 225 | 150 | 75 | 0 |
|  | **Snack** | 150 | 112.5 | 75 | 37.5 | 0 |
| **Bolus ml for Required Intake 50%** | **Main Meal** | 200 | 150 | 100 | 50 | 0 |
|  | **Snack** | 100 | 75 | 50 | 25 | 0 |

*Nasogastric Enteral Nutrition Support*

Overnight NG feeding is used for patients unable to tolerate nutrition supplements orally and is presented as an option available at any time during the admission should it be required. For example, the NG tube can be a supportive measure to help patients manage compensatory purging behaviors during re-feeding. An initial volume of 600 ml of a 1.5 kcal/mL nutritionally complete and balanced, fibre-free, liquid tube feed is administered overnight for 10 hours. Overnight NG feeds are increased by 200 ml on alternate days from day 9 of the protocol until the target volume of 1000 ml is reached. Patients are still required to consume meals and snacks consistent with the re-feeding protocol, however three snacks replace the three oral supplements scheduled at snack times on days 1-6 of the protocol, and snack goal intake is consistent with the meal proportion. For example, 50% meals and snacks on days 1 and 2 of the protocol. Patients are limited to one bolus daily from Day 13 of the protocol ***(Supplementary Table S1*)**, to reduce the reliance of ONS over food. Additional boluses required are administered via the NG tube at a higher rate with the rationale of food being incentivised over the NG feed.

*Management of Complications*

Patients who experience two or more episodes of hypoglycaemia in 24-hours commence continuous NG feeding to stabilise blood glucose levels. A 1.5 kcal/ml nutritionally complete and balanced, fibre-free, liquid tube feed is initiated at 30 ml/hr. Meal and snack increments on alternate days are consistent with the re-feeding protocol. NG feed increments follow by 10 ml/hr on alternate days to 40 or 50 ml/hr dependent on length of time continuous feeding is required. NG tubes are flushed with 50 ml water every 4 hours. Once blood glucose levels have remained stable for a 5-day period, the NG feed is converted to a 10-hour overnight feed. In the event of any symptoms of re-feeding syndrome indicated by the presence of medical risk factors (hypophosphatemia, hypomagnesaemia, hypokalaemia, hyponatraemia, reduced thiamine levels and oedema) the rate of re-feeding is reviewed and modified in line with existing clinical practice guidelines (7).

*ED Presentations Requiring Weight Maintenance*

The re-feeding protocol may also be used for patients with EDs who are admitted primarily to manage compensatory behaviours, such as purging in patients with BN. Initial goal intake is based on pre-admission intake with final intake goal of three meals and three snacks, which may be further modified to meet individual requirements.

**Meal Planning**

The four goals of meal planning are to 1) plan a regular and adequate intake to support individual re-feeding goals, 2) promote a varied intake of meals and snacks, 3) provide nutrition education using a balanced approach to eating and inclusion of all food groups described in the Australian national guide to healthy eating and tailored for people with EDs (8) and 4) challenge the avoidance of foods and food groups.

*Regular and adequate eating* is facilitated through patients completing menus with dietitian support three times per week on the same day weight measurements are recorded. Initially meal plan changes are consistent with the re-feeding protocol, and beyond Day 12 of admission, after assessment of the patient’s progress with weight restoration. Standard meal plans for females and males (Supplementary ***Table S3***) are adapted by the dietitian to accommodate individual dietary requirements. For example, gluten free meals and snacks for diagnosed coeliac disease and nut free meals and snacks for diagnosed nut allergies. Only whole milk and dairy products are provided.

*Insert table 3. Baseline Daily Meal Plans for Females and Males*

*Variety* is promoted through the provision of choice and the use of boundaries, and the rationale for this is clearly discussed with patients. Completing menus in two- or three-day blocks ensures that a variety of different meals and snacks are chosen for that period. For example, both meat and lacto-ovo-vegetarian options are available at each main meal. At breakfast there are a variety of protein options available on different days from the meat and meat alternatives food group, including bacon, sausages, eggs, and baked beans. To ensure two servings from this group are included daily by lacto-ovo-vegetarians, one of the items from this group is a required choice as lacto-ovo-vegetarian hospital dinner options often only provide protein from the dairy and dairy alternatives food group. At lunch a minimum of two different sandwich fillings over two days of menus is required. At dinner, the dinner rolls are limited to one of the two or more grain serves required, to promote inclusion of different grain or potato choices. At afternoon tea, different bakery items or cookies are provided daily on a two-week menu cycle. Four different snack items over two days, at morning tea and supper, are required to be chosen from a varied list of options. Beyond the minimum requirements for variety outlined, patients are also encouraged to work to increase variety and challenge individual food avoidance with support from the dietitian.

*Nutrition Education* occurs informally during group meal planning sessions three times per week and formally through weekly dietitian led nutrition education groups. Nutrition groups are interactive and include quizzes, discussion, and a variety of audiovisual media to present evidence-based nutrition information. Topics covered include starvation syndrome, medical complications of EDs, what to expect during re-feeding, metabolism and changes during starvation and re-feeding, digestion, returning to normal eating, food groups, energy nutrients, restoring to a healthy weight and meal planning for home.

*Avoided foods/food groups are challenged* through the meal planning and the requirements described above to ensure adequate intake and promote variety. A weekly social eating group provides an opportunity for patients to experience foods commonly eaten in a social setting such as pizza, burgers, chocolate, and ice cream. The nutritional and social value of foods is discussed during the social eating group and ED cognitions are challenged.

**Operational factors supporting the re-feeding protocol**

To implement this higher energy re-feeding protocol effectively and safely and to optimise patient readiness for discharge from hospital, it is imperative to consider the following additional integral operational factors.

*A cohesive multidisciplinary team approach with an emphasis on strong therapeutic engagement* promotes efficient weight and nutritional restoration, ensures patient safety, and minimises patient physical and psychological discomfort. Our team consists of a single-psychiatrist ED program lead, specialist nurse coordinator, dietitian, psychiatric registrar, resident medical officer, clinical psychologist, ED trained nursing staff and a peer support worker. Medical monitoring facilitates early identification and treatment of medical instability. Prophylactic phosphate, magnesium and multivitamins are administered to prevent re-feeding syndrome. Ongoing medical and psychological review by all treatment team members optimizes management of the psychological and physical effects of re-feeding including anxiety, insomnia, nausea, and constipation. A collaborative approach is supported through twice weekly team meetings, weekly patient, family, and carer meetings, with additional patient reviews as required, to support patients to manage their symptoms and compensatory behaviors or urges that arise. Treatment is patient-centered and incorporates trauma-informed care.

*A pre-admission assessment and comprehensive discharge planning promote a seamless transition through the continuum of inpatient to ED day program or outpatient care.* The pre-admission assessment by the specialist nurse coordinator identifies and prioritises patients who have the capacity to engage in voluntary treatment (Supplementary ***Figure S2***) and provides potential patients with information relating to the admission and ED program, enabling them to make an informed decision about their admission. Discharge planning begins on the day of admission. Individual outpatient teams and therapy programs are identified, and referrals made, to support patients in their recovery beyond hospital. A detailed discharge summary is provided to the outpatient treatment team that includes the primary healthcare physician, psychologist, and dietitian.

*A stage-based ED program* provides patients with boundaries around behavioral expectations, and markers to identify progress through the program. All mealtimes are supervised by nursing staff that provide meal support to model normal eating, encourage patients to eat their required intake and to monitor and manage compensatory behaviors such as hiding food. Main meals are limited to 25 min and snacks to 10 min. For 30 min after main meals, nursing staff provide support and supervision in the form of distraction groups. For patients requiring additional support to manage compensatory behaviours following meals and snacks, individual plans are created in collaboration with their treatment team. Increased independence at mealtimes is facilitated once patients are confidently managing their entire meal plan and reflects the patient’s own supports available following hospital discharge. ED specialist nursing care focuses on managing distress and discomfort and on the development of distress tolerance plans for use both in hospital and following discharge. Evidence-based group therapy for EDs is provided twice daily in 1-1.5 hr sessions, and includes cognitive behavioural therapy-enhanced, dialectical behavioural therapy, goal setting, inter-personal therapy, nutrition education and social eating groups, and peer support. The program is reviewed annually to facilitate adaption and incorporation of new research. The staged structure of the ED program and weekly group timetable are summarised in Supplementary ***Tables S4 & S5***

*ED-specific knowledge and skills* are required by the dietitian to provide safe and effective care for people with eating disorders. Training standards and ED credentialing have been developed to provide a foundation from which dietitians can develop competent practice and require that dietitians engage in ongoing professional development and supervision pertinent to their setting (9–11).

*ED Program evaluation* occurs annually through audit of patient weight and psychological change from admission and discharge. Variables collected include diagnosis, age, length-of-stay, weight, BMI, and psychosocial test scores (ED Examination Questionnaire, Depression Anxiety Stress Score -21, Clinical Impairment Assessment for EDs and AN and BN Stage of Change Questionnaires). Dietitians working with people with EDs in the inpatient setting are well placed to contribute to the ongoing improvement of treatments and outcomes for people with EDs by embedding audit and research into their clinical practice (12).

The re-feeding protocol in this ED program is effective in achieving its goal of 1.5-2 kg/week positive weight change for underweight adolescents and adults with AN and adults with ARFID and provides a baseline from which to evaluate this ED program service.

**List of abbreviations**

**ED:** Eating Disorder

**AN:** Anorexia Nervosa

**BN:** Bulimia Nervosa

**ARFID:** Avoidant Restrictive Food Intake Disorder

**ONS:** Oral Nutrition Supplements

**BMI:** Body Mass Index

**NG:** Nasogastric

**Supplementary References**

1. Drysdale C, Matthews-Rensch K, Young A. Further evidence to throw caution to the wind: outcomes using an assertive approach to manage refeeding syndrome risk. Eur J Clin Nutr. 2021;75(1):91–8.

2. Friedli N, Stanga Z, Sobotka L, Culkin A, Kondrup J, Laviano A, et al. Revisiting the refeeding syndrome: Results of a systematic review. Nutrition. 2017;35:151–60.

3. National Institute for Health and Care Excellence. Nutrition support for adults: oral nutrition support, enteral tube feeding and parenteral nutrition. 2006.

4. Garber AK. A few steps closer to answering the unanswered questions about higher calorie refeeding. J Eat Disord. 2017;5(1):4–6.

5. Haas V, Kohn M, Körner T, Cuntz U, Garber AK, Le Grange D, et al. Practice-Based Evidence and Clinical Guidance to Support Accelerated Re-Nutrition of Patients With Anorexia Nervosa. J Am Acad Child Adolesc Psychiatry. 2020;1–2.

6. CDC National Center for Health Statistics. National Health and Nutrition Examination Survey. [cited 2024 Apr 13]. Growth Charts. Available from: https://www.cdc.gov/nchs/nhanes/index.htm

7. Hay P, Chinn D, Forbes D, Madden S, Newton R, Sugenor L, et al. Royal Australian and New Zealand College of Psychiatrists clinical practice guidelines for the treatment of eating disorders. Australian and New Zealand Journal of Psychiatry. 2014;48(11):977–1008.

8. Hart S, Marnane C, McMaster C, Thomas A. Development of the “Recovery from Eating Disorders for Life” Food Guide (REAL Food Guide) - a food pyramid for adults with an eating disorder. J Eat Disord. 2018;6:6.

9. Heruc G, Hart S, Stiles G, Fleming K, Casey A, Sutherland F, et al. ANZAED practice and training standards for dietitians providing eating disorder treatment. J Eat Disord. 2020;8(1):1–10.

10. Jeffrey S, Heruc G. Balancing nutrition management and the role of dietitians in eating disorder treatment. J Eat Disord. 2020;8(1):8–11.

11. Heruc G, Hurst K, Casey A, Fleming K, Freeman J, Fursland A, et al. ANZAED eating disorder treatment principles and general clinical practice and training standards. J Eat Disord. 2020;8:1–9.

12. InsideOut. National Clinical Minimum Data Set & Data Capture at the Point of Care [Internet]. 2022. Available from: https://insideoutinstitute.org.au/projects/

**Supplementary Table S3. Baseline Daily Meal Plans for Females and Males**

| **Food Groups and Serve Sizes** | **Required Serves** | |
| --- | --- | --- |
|  | **Females** | **Males** |
| **Breakfast** | | |
| **Grains** e.g. 2 Weetabix *or* 1 cup cereal flakes *or* 1 slice toast *or* ¼ cup muesli  ***or* grains + 1 meat /meat alternative** e.g. 2 eggs *or* 2 rashers of bacon *or* 1 cup baked beans | 3  *or*  2 + 1 | 4  *or*  3 + 1 |
| **Dairy** or soy based equivalent milk e.g. 1 cup milk *or* 200g pot yoghurt | 1 | 1 |
| **Fruit** e.g. 1 whole piece of fruit, ½ cup fruit juice *or* 1 cup diced or canned fruit | 1 | 1 |
| **Fats & Oils** e.g. 1 teaspoon margarine *or* butter | 1-2 | 2-3 |
| **Morning Tea** | | |
| **Snack** e.g. nut bar, muesli bar, cheese or hummus & crackers, nuts or trail mix, raisin toast *or* chocolate bar | 1 | 1 |
| **Lunch** | | |
| **Grains and potato** e.g. 1 slice bread, ½ cup potato, ½ cup rice, pasta, couscous, quinoa *or* other grain | 3 | 4 |
| **Meat or Dairy** e.g. palm-size portion of ham, chicken, tuna, 2 eggs *or* 2 large slices cheese | 1 | 1.5 |
| **Fats & Oils** e.g. 1 teaspoon margarine, butter, mayonnaise *or* oil based salad dressing | 2 | 3 |
| **Vegetables or salad** ½ - 1 cup | 0-1 | 0-1 |
| **Fruit** e.g. 1 whole piece of fruit*,* ½ cup fruit juice, 1 cup diced or canned fruit *or* other **dessert** | 1 | 1 |
| **Afternoon Tea** | | |
| **Bakery Item** e.g. scone & margarine *or* butter, slice of cake *or* 3 small cookies | 1 | 1 |
| **Dinner** | | |
| **Grains and potato** e.g. 1 slice bread, ½ cup potato, ½ cup rice, pasta, couscous, quinoa *or* other grain | 3 | 4 |
| **Meat or Dairy** e.g. palm-size portion of ham, chicken, tuna, 2 eggs *or* 2 large slices cheese | 1 | 1-2 |
| **Fats & Oils** e.g. 1 teaspoon margarine, butter, mayonnaise *or* oil based salad dressing | 1 | 1-2 |
| **Dairy** e.g. 200g pot yoghurt *or* ½ serve dairy *and* 1 serve of fruit e.g. 1 small pot custard *or* 1 scoop ice-cream *and* 1 cup fruit salad | 1 | 1 |
| **Supper** | | |
| **Snack** e.g. 2 crumpets, 3 pikelets *or* 1 English muffin with butter or margarine *or* 200g pot of yoghurt *or* chips | 1 | 1 |

**Supplementary Table S4. Summary of** **Inpatient ED Program Stages and Management**

| **Stage** | **1** | **2a / 2b** | **3** |
| --- | --- | --- | --- |
| **Nutritional**  **Intake** | - Completes menus in consultation with dietitian - Consumes meals, snacks and oral nutritional supplements as prescribed by dietitian - Nasogastric feed, if required, as prescribed by dietitian - Consumes meals within 25 minute and snacks within 10 minute time limit - All meals and snacks supervised by nursing staff and intake recorded - Attends rest period in group room supervised by nursing staff for 30 minutes after main meals - Attends additional rest period as required semi-supervised near nursing station or in group room | - As stage 1 with increasing independence at snack then mealtimes, individualized in relation to patient progress with weight goals and eating confidence | - Meal planning through menu completion with support of dietitian if required - Meals and snacks consumed independently - Patient completes food intake charts for discussion with the dietitian - No supervised rest period required |
| **Fluid**  **Intake** | - 1 cup water with each meal or snack - 1 liter bottle water during day/night - 3 hot drinks daily after snack time | - As stage 1 | - Fluid intake unsupervised |
| **Activity** | - Walk between bedroom, dining room and group rooms only | - **Stage 2a** - attend 30 minutes movement group daily supervised by exercise physiologist | - Encouraged to attend daily movement group & minimize physical activity outside group - Activity unsupervised |
| **Bathroom** | - Locked initially until risk of purging, compulsive exercise, assessed - Supervised by nursing staff to support management of compensatory purging or exercise - Locked overnight if NG feeding - Unlocked if none of above apply | - Twice weekly assessment of purging, compulsive exercise risk in consultation with patient - Unlocked when compensatory behaviors managed and weight goals progressing | - Unlocked |
| **Weight** | - Blind weigh 3 x weekly (Mon/Wed/Fri) - Team discuss minimum weight target for admission with patients requiring weight restoration - Target weight restoration 1.5-2.0Kg/week   *Note: Weight increase in first week may be greater* *due to fluid changes* | - As stage 1 | - Weighing frequency reduced to 1 - 2 times weekly if weight restored - Patients may choose to view plotted BMI weekly in consultation with ED team |
| **Leave** | - No leave | - **Stage 2a** - approved leave permitted for appointments outside of group time - **Stage 2b** - increasing weekend leave in-between meals and snacks, then leave for snacks, then meals, individualized to patient progress with weight goals and eating confidence - Approved leave permitted for special circumstances during week | - Approved leave over meal and snack times permitted |
| **Medical**  **Monitoring** | **On admission:**   - Bloods - FBC, LFTs, UEC, Ca^2+^, Mg^3+^, PO_4_^3-,^ Vit D, Iron studies, Vit B12, Folate - ECG - Consider medical consult if BMI <14 - Vital signs including sitting/standing HR & BP - BGLs   **1^st^ 14 days:** 3 x weekly Mon/Wed/Fri or daily if high risk of re-feeding syndrome* monitor closely   - Bloods - LFTs, UEC, Ca ^2+^, Mg^3+^, PO_4_^3-^ - Vital signs including sitting/standing HR & BP - review daily reducing frequency to daily once in normal parameters f - BGLs 4 times daily - 2 hours after main meals (before snacks) and at 2 am until within normal parameters for 5 days   **Weekly thereafter** Mondays:   - FBC - Bloods as above - Consider: Bone Scan if amenorrhea > 6 months | - As stage 1 | - If all results within normal parameters, no further monitoring |
| **Medications** | - Complete Multivitamin-mineral supplement x 2 tablets OD mane - Phosphate Sandoz 500 mg x 2 mane for 2 weeks - Calcium Carbonate 600 mg x 2 tablets nocte - Thiamine 300 mg 1 OD - Replace abnormal electrolytes, vitamins as clinically indicated e.g., Magnesium Aspartate Dihydrate 1 tab = 37.4 mg Magnesium, cholecalciferol 1 capsule = 1000 iu - Probiotic capsule x 1 = 32 billion CFU mane - Consider: Non-stimulant fiber/laxatives e.g., Benefibre 2 tsp. BD, Movicol 1- 2 BD - Consider: Short acting benzodiazepine Lorazepam 0.5-1.0 mg prn or before main meals for high distress levels – to be ceased prior to discharge | - As stage 1 | - As medically required |
| **Extra**  **Bed rest:**  **if medically**  **compromised** | - Sit or lie down on bed/chair - Attend all meals, snacks, rest period, groups in wheelchair - ½ hourly visual observations – bedroom door to be left open - Call bell to be used to request nurse assistance - Shower once daily for 10 minutes - Assistance provided by nursing staff and family for laundry and other physical tasks | - Not Applicable | - Not Applicable |
| **Stage Progression** | Progression through stages 1 - 3 occurs following assessment of weight restoration, dietary intake and patient’s engagement with ED program. | | |

**Supplementary Table S5.** **Summary of ED Inpatient groups**

| **Day/Time** | **Monday** | **Tuesday** | **Wednesday** | **Thursday** | **Friday** | **Weekends** |
| --- | --- | --- | --- | --- | --- | --- |
| **07.30-08.30** | Supervised **Breakfast** and post meal **Rest Period** with **Nursing Staff** | | | | | |
| **09.00-09.30** | **Mindful movement:** walk, Pilates, stretch, restorative yoga or education group with **Exercise Physiologist**  **Stages 2a /b & 3 only** | | | | | |
| **10.00-10.15** | Supervised **Snack** with **Nursing Staff** | | | | | |
| **10.30-11.30** | **Goal Setting** with  **Psychologist** | **Nutrition** group with **Dietitian** | **Social Eating Group** with **Dietitian** and **Occupational Therapist** | **CBT-e** with **Psychologist** | Patient, Family, Team **Review Meeting,** concurrent with **Nursing Staff** led **Distraction group** | **General** groups e.g. distraction, led by **Nursing Staff** as available |
| **12.00-13.00** | Supervised **Lunch** and post meal **Rest Period** with **Nursing Staff** | | | | | |
| **13.15- 14.30** | **IPT** with **Psychologist** | **Process group** with **ED Nurse Specialist** | **DBT** with **Psychologist** | **CBT-e** with **Psychologist** | **Recovery Group** with **Peer Support Worker** | **General groups** e.g. distraction, led by **Nursing Staff** as available |
| **15.00-15.15** | Supervised **Snack** with **Nursing Staff** | | | | | |
| **18.00- 19.00** | Supervised **Dinner** and post meal **Rest Period** with **Nursing Staff** | | | | | |
| **20.00- 20.15** | Supervised **Snack** with **Nursing Staff** | | | | | |

CBT-e - Cognitive Behavioral Therapy-enhanced for EDs, DBT - Dialectical Behavioral Therapy, IPT - Inter-Personal Therapy.

**Declarations**

**Ethics Approval and Consent to Participate:** Not applicable.

**Consent for Publication:** Not applicable.

**Availability of Data and Materials:** The datasets used and/or analysed during the current study are available from the corresponding author on reasonable request.

**Competing Interests:** The authors declare they have no competing interests.

**Funding:** Hollywood Private Hospital Research Foundation provided a small grant award to support publication of this manuscript

**Author’s Contributions:** FS and US contributed to the conception and design of the re-feeding protocol and eating disorder program supporting the protocol; FS and EJ contributed to the concept of the paper and FS and EJ drafted the manuscript. All authors critically revised the manuscript, agree to be fully accountable for ensuring the integrity and accuracy of the work, and read and approved the final manuscript.

**Acknowledgements:** Not applicable.
