# Supplementary material for: A prospective observational study examining weight and psychosocial change in adolescent and adult eating disorder inpatients admitted for nutritional rehabilitation using a high-energy re-feeding protocol
Source: J Eat Disord. 2024 May 14;12:58. doi: 10.1186/s40337-024-01015-x (PMC11094855; doi:10.1186/s40337-024-01015-x)
Supplement: Supplementary file 2 — (pdf 36 KB) [file 40337_2024_1015_MOESM2_ESM.pdf]

NUTRITION ASSESSMENT

Medical History - ED diagnosis, other diagnoses, medications		
**Social History - school/work/study, living situation, support people**		
**Influences - family/peers attitudes, diet culture, social media, family ED History**		
**ED History - age of onset, weight & shape concern, dieting behaviour, treatment**		
**Menstrual History - age at menarche, cycle regularity**		
Bone scan (if LMP >6mths ago)? Yes / No Hormonal contraception? Yes / No		
**Community care team - clinician's name and location**		
Psychologist:		
Dietitian:		
Psychiatrist:		
Primary Healthcare Physician:		
**Weight History**		
Highest wt:	Lowest wt:	The ED's ideal wt:
Date:	Date:	What patient believes is their healthy weight:
Weight change over previoua 6/12, 3/12, 1/12:		Frequency of weighing and by whom:

Recent intake	
Breakfast	Afternoon
Morning	Dinner
Lunch	Supper
NutritionaL Supplements:	Prescribed? Yes / No
Water & other drinks:	
Alcohol:	
Diagnosed food allergies & intolerances:	
**Restrictive behaviours - diet apps, calorie counting, avoided foods/groups, skipping meals**	
**ED rules & rituals, mealtime behaviours**	
**Other ED behaviours - frequency, triggers, age of onset**	
Binge eating - subjective / objective:	
Self-induced vomiting:	
Chewing & spitting:	
Laxative misuse:	
Other eg. Diet pills, diuretics:	
**Physical activity (past and current)**	
Exercise to make up for eating? Yes / No	Does exercise feel compulsive? Yes / No
**Patient insights**	
Perceived benefits of ED:	
Costs of ED (physical, psycho-social):	
Motivators for change:	
Patient's goals for admission:	
**Notes**	
DIETITIAN NAME / SIGNATURE:	DATE:
