# Supplementary material for: A prospective observational study examining weight and psychosocial change in adolescent and adult eating disorder inpatients admitted for nutritional rehabilitation using a high-energy re-feeding protocol
Source: J Eat Disord. 2024 May 14;12:58. doi: 10.1186/s40337-024-01015-x (PMC11094855; doi:10.1186/s40337-024-01015-x)
Supplement: Supplementary file 3 — (pdf 68 KB) [file 40337_2024_1015_MOESM3_ESM.pdf]

Inpatient Eating Disorder (ED) Program Treatment
Admission Criteria
1. Primary diagnosis of ED<br>2. Minimum age 16<br>3. BMI > 13 kg/m^2<br>4. Meet Country X eating disorder admission criteria<br>5. Display capacity to engage in voluntary ED treatment
Prioritise
1. Lower BMI<br>2. Medical instability<br>3. Age 16 - 18 years<br>4. First ED presentation<br>5. Short duration of illness, or longer duration of illness and no previous inpatient ED Program treatment
